# Commentary: Vasa vasorum dysfunction and acute aortic syndromes: When guidelines do not follow the evolution of knowledge

**DOI:** 10.1016/j.xjon.2020.12.015

**Published:** 2020-12-29

**Authors:** Antonio M. Calafiore, Kostas Katsavrias, Massimo Di Marco, Stefano Guarracini, Michele Di Mauro

**Affiliations:** a Department of Cardiovascular Sciences, Gemelli Molise, Campobasso, Italy; b Department of Cardiac Surgery, Henry Durant Hospital, Athens, Greece; c Department of Cardiology, “Santo Spirito” Hospital, Pescara, Italy; d Department of Cardiology, “Pierangeli” Hospital, Pescara, Italy; e Cardio-Thoracic Surgery Unit, Heart and Vascular Centre, Maastricht University Medical Center, Cardiovascular Research Institute Maastricht, Maastricht, the Netherlands

**Figure:**
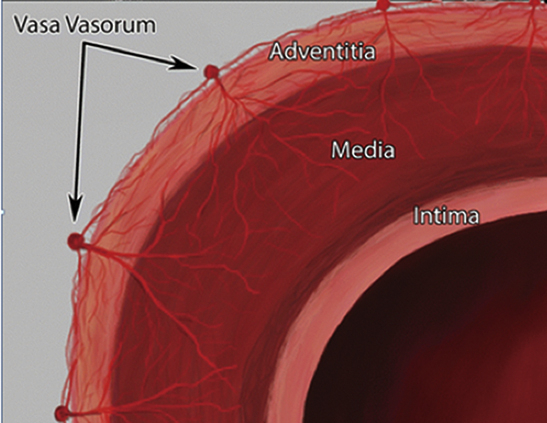
Large systemic artery and its vasa vasorum network. The vasa vasorum of the ascending aorta originates from the coronary arteries and from the brachiocephalic arteries, and that of the descending aorta originates from the intercostal arteries. It has been observed that vasa are present in the media of those animals in which the vessel wall thickness is >0.5 mm, with the exception of coronary arteries. Another determinant of the presence of vasa vasorum is the luminal oxygen tension: large veins (or the pulmonary artery) with thin walls but low luminal oxygen tension are supplied by a dense network of vasa.

**Central Message**

Vasa vasorum dysfunction can be the unpredictable event that causes intramural hemorrhages and intimal tears, causing acute aortic syndromes even with a mild or moderate increase in aortic size.

See Article page 30.

The aorta is a very strong structure. Aortic rupture does not occur if the intraluminal pressure ranges from 790 to 2070 mm Hg.^1^ Robertson and Smith, injecting water into the media of 42 fresh human aortas, found that the lowest pressure required to exceed the cohesive strength of the media was 273 mm Hg and the highest was 975 mm Hg, with a mean of 566 mm Hg.^2^ Therefore, the aorta is highly resistant to rupture or dissection.

In this issue of the *Journal*, Haverich and Boyle^3^ repropose an appealing unifying theory on the genesis of aortic intramural hematoma (AIMH) and aortic dissection (AD). Vasa vasorum dysfunction is the link between these entities, which are seen as progression of one to the other. Rupture and bleeding of the vasa vasorum into the media is the cause of AIMH. It can remain limited to the thickness of the aorta or cause an intimal tear, which is at the basis of classic AD.

The vasa vasorum fill during diastole as in the coronary circulation. Thus, an increase in arterial diastolic pressure in the host vessel results in reduced perfusion,^4^ which can cause vessel wall hypoxia and neoangiogenesis, with the neovessels more fragile and prone to bleed. Hypertension also can reduce blood flow by distortion or compression of the vasa, generating changes in the walls of the vasa vasorum with critical ischemia and necrosis of the media. Other factors (eg, inflammation) can induce aberrant and adverse remodeling of the aortic wall, including smooth muscle cell loss in the media and extracellular matrix degradation in the media and the adventitia. The consequence is chronic dilation of the aorta, but an acute aortic syndrome (AAS) can superimpose at any moment.

The guidelines for prophylactic surgery of ascending aortic aneurysms include only the aortic size, with a cutpoint of 55 mm in non-Marfan nonbicuspid valve aortas.^5^ Tozzi and colleagues^6^ found that 87.7% of patients with AD had a preoperative aortic size <45 mm, and that a threshold of 55 mm excluded ∼99% of patients with AD from prophylactic surgery. The same findings have been reported by others.^7^ Aortic size is only a surrogate marker, often unreliable, of catastrophic events, and the guidelines need to recognize the difficulty of linking aortic size to AAS. An update of the natural history of ascending aortic aneurysms from the Yale team found that the hinge point for rupture or dissection was reduced from 60 mm in an earlier study of 230 patients to 52.5 mm in a study of 3400 patients.^8^ Other unpredictable events, including vasa vasorum dysfunction, can occur at any time. Genetic screening in individuals with nonsyndromic aortic aneurysms remains a work in progress, and clinical implications are not widely accepted.^9^^,^^10^ As it is impossible to foresee which aorta at which size could undergo an acute event, reducing the aortic size for prophylactic surgery is our only tool for treating more patients before AAS occurs.
